# Diabetes: A Multifaceted Disorder

**DOI:** 10.3390/biomedicines10071698

**Published:** 2022-07-14

**Authors:** María Grau, Carles Pericas

**Affiliations:** 1 Serra Húnter Fellow, Department of Medicine, University of Barcelona, 08036 Barcelona, Spain; carlespericas@ub.edu; 2 Biomedical Research Consortium in Epidemiology and Public Health (CIBERESP), 08036 Barcelona, Spain; 3 August Pi i Sunyer Biomedical Research Institute (IDIBAPS), 08036 Barcelona, Spain

Diabetes is a chronic disease associated with increased morbidity and mortality from cardiovascular diseases cancer, chronic obstructive pulmonary disease, and kidney or liver disease [1,2]. Despite the fact that aging is the main risk factor for the onset and progression of type 2 diabetes mellitus (the most common type of diabetes), in recent decades, age-standardized all-cause mortality in the population with type 2 diabetes has fallen more than in the general population [3]. However, age-specific data indicate that this trend in the total diabetes population is predominantly influenced by trends in those aged 80 or more years. The figures observed in those younger than 80 years indicate that improvements in the management of diabetes and its complications may not have translated into a direct prevention of premature deaths related to type 2 diabetes [4].

This Special Issue of *Biomedicines* presents a compilation of high-quality scientific evidence aimed at unraveling the molecular mechanisms involved in the association between diabetes and comorbidities and at describing their clinical and therapeutic implications. The issue has been structured accordingly and subdivided in three sections.

The first section focuses on diabetes mellitus itself; the second focuses on the complications of such a disorder—particularly nephropathy, polyneuropathy, and diabetic retinopathy—and the third one presents the consequences of COVID-19 in the population with diabetes.

The first section starts with a narrative review about the molecular mechanisms implied in the failure of pancreatic β-cell failure [5]. The review is followed by a computational model aimed to analyze the effects of hyperlipidemia, particularly free fatty acids, on pancreatic beta cells and insulin secretion [6]. After, two experimental studies, the first examines the role of tyrosine isoforms ortho- and meta-tyrosine in the development of insulin resistance [7]. The second, conducted in a mouse model, focuses on the impairment of the insulin signaling pathway in the placenta during pregnancies complicated by maternal obesity and gestational diabetes mellitus [8]. Additionally, four studies conducted in human samples have been included. The first one is a cross-sectional study, which assessed the relationship between adipokines, and their ratio with obesity and diabetes [9]. The second one is an intervention study, which evaluated a 13-week personalized lifestyle intervention in newly diagnosed type 2 diabetes mellitus. The results showed an improvement in type 2 diabetes mellitus parameters compared with patients who received usual care [10]. The final two studies are narrative reviews devoted to the treatment of diabetes mellitus. One suggests that the nuclear factor-kappa B could be a target for an anti-inflammatory strategy in preventing and treating diabetes when immunoglobulin-free light chain is modified [11]. The second focuses on the role of β-cell mass/proliferation pathways, dysregulated in diabetes, in treating and reversing diabetes as well as the current therapeutic agents studied to induce β-cell proliferation [12].

In the second section, three manuscripts show the broad spectrum of pathologies related with diabetes. First, an experimental study in a mouse model presents the detection of MORG1 expression, a scaffold protein, as a promising strategy to reduce lipid metabolic alterations in diabetic nephropathy [13]. Regarding the other studies, conducted in human samples, Fujishiro et al. evaluated the impact of plasma xanthine oxidoreductase activity on the mechanisms of distal symmetric polyneuropathy development [14], and Boned-Murillo et al. performed a systematic review to analyze the current applications of optical coherence tomography angiography and to provide an updated overview on its role in the evaluation of diabetic retinopathy [15].

Finally, COVID-19 also has a space in our Special Issue, with two scientific works. The first article is a narrative review, which provides an overview of the most recent studies that determine type 2 diabetes mellitus as a risk factor and link it to poor prognosis of COVID-19 [16]. The second one assessed how alpha-2 macroglobulin, apolipoprotein A1, and haptoglobin are associated with the risk of liver fibrosis, inflammation, and COVID-19 in patients with non-alcoholic fatty liver disease with or without type 2 diabetes mellitus [17].

The variety of updated topics included from different approaches shows the need for more efficient preventive activities to reduce the incidence of this disease and its related complications.

On one final note, it is important to point out that diabetes is a worldwide public health problem that can be explained by the classic model of the determinants of health. This model shows how individual lifestyles are embedded in social norms and networks and in living and working conditions, which in turn are related to the wider socioeconomic and cultural environment [18]. Therefore, success in preventing diabetes mellitus and its associated complications not only depends on the individual but also on social and community networks as well as general socioeconomic, cultural, and environmental conditions [19]. Indeed, the final aim is to create conditions that ensure good health and social care for an entire population through the development and implementation of preventive strategies, promotion of healthy lifestyles, protection from diseases, and the design of targeted screening strategies. This aligns with the United Nations Sustainable Development goal number 3, good health and well-being, which includes the achievement of universal health coverage and access to quality essential healthcare services. In addition, this also has a direct link to goal number 10, which focuses on reducing inequalities within and among countries [20].

Additionally, we have analyzed the gender balance and authors’ nationalities in this Special Issue. Although 62% of the first authors are women, after analyzing the percentage of senior authors by gender, this figure dropped to 38%. The authors of the works included are from nine different countries, guarantying an international representation.
